# A Sexual Shift Induced by Silencing of a Single Insulin-Like Gene in Crayfish: Ovarian Upregulation and Testicular Degeneration

**DOI:** 10.1371/journal.pone.0015281

**Published:** 2010-12-09

**Authors:** Ohad Rosen, Rivka Manor, Simy Weil, Ohad Gafni, Assaf Linial, Eliahu D. Aflalo, Tomer Ventura, Amir Sagi

**Affiliations:** 1 Department of Life Sciences, Ben-Gurion University of the Negev, Beer-Sheva, Israel; 2 The National Institute for Biotechnology in the Negev, Ben-Gurion University of the Negev, Beer-Sheva, Israel; Temasek Life Sciences Laboratory, Singapore

## Abstract

In sequential hermaphrodites, intersexuality occurs naturally, usually as a transition state during sexual re-differentiation processes. In crustaceans, male sexual differentiation is controlled by the male-specific androgenic gland (AG). An AG-specific insulin-like gene, previously identified in the red-claw crayfish *Cherax quadricarinatus* (designated *Cq-IAG*), was found in this study to be the prominent transcript in an AG cDNA subtractive library. In *C. quadricarinatus*, sexual plasticity is exhibited by intersex individuals in the form of an active male reproductive system and male secondary sex characters, along with a constantly arrested ovary. This intersexuality was exploited to follow changes caused by single gene silencing, accomplished via dsRNA injection. *Cq-IAG* silencing induced dramatic sex-related alterations, including male feature feminization, a reduction in sperm production, extensive testicular degeneration, expression of the *vitellogenin* gene, and accumulation of yolk proteins in the developing oocytes. Upon silencing of the gene, AG cells hypertrophied, possibly to compensate for low hormone levels, as reflected in the poor production of the insulin-like hormone (and revealed by immunohistochemistry). These results demonstrate both the functionality of *Cq-IAG* as an androgenic hormone-encoding gene and the dependence of male gonad viability on the Cq-IAG product. This study is the first to provide evidence that silencing an insulin-like gene in intersex *C. quadricarinatus* feminizes male-related phenotypes. These findings, moreover, contribute to the understanding of the regulation of sexual shifts, whether naturally occurring in sequential hermaphrodites or abnormally induced by endocrine disruptors found in the environment, and offer insight into an unusual gender-related link to the evolution of insulins.

## Introduction

Understanding sexual shifts and differentiation in both vertebrates and invertebrates is of major interest, particularly given our changing environment. In vertebrates, such as fish, the regulation of such processes can be attributed not only to genetics but also to the effects of hormones and/or environmental factors [1], [2]. In invertebrates, too, diverse factors are involved in the regulation of sexual differentiation. For example, in arthropods, such as *Drosophila melanogaster*, gender-biased alternative splicing constitutes an important component of sexual differentiation [3]. Although crustaceans are also considered to be Arthropoda and even believed to be potential ancestors of insects [4], these ancient marine organisms demonstrate regulation of sexual differentiation that is significantly different from that of insects. In Crustacea, the process is governed by the androgenic gland (AG) [5], an endocrine gland restricted to males that secretes male sex hormone(s) [6]. It is believed that female sexual development and secondary characteristics are exhibited only in the absence of the AG, thereby establishing femaleness as a matter of default, negatively regulated by the AG [7]. In crustaceans, AG involvement in inducing masculinity has been thoroughly investigated in several species [8], [9]. In the Australian red-claw crayfish, *Cherax quadricarinatus*, the implantation of AGs into immature females led to the replacement of female characteristics with male traits, as well as to the cessation of vitellogenesis [10]–[12].

The vast array of observed AG-associated effects has been attributed to an AG-borne hormone. Three orthologs of this putative AG hormone have been isolated from three species of isopods [13], [14]. Recently, two uniquely AG-expressed genes were revealed using AG cDNA libraries prepared from the decapods, *C. quadricarinatus* (*Cq-IAG*) and the freshwater prawn, *Macrobrachium rosenbergii* (*Mr-IAG*) [15], [16]. Structurally, both sequences resemble genes of the insulin-like superfamily [15]. Although evidence supporting a role for insulin in sexual differentiation has been previously documented [17], the unique instance of a gender-specific insulin-like gene has thus far been reported exclusively in crustaceans. Thus, it is possible that insulins may have evolved in the context of regulation of sexual differentiation and not exclusively on the background of metabolism and growth.

AG hormone ineffectiveness, whether induced by AG ablation or resulting naturally from insufficient levels of secreted hormone(s), can account for hermaphroditism, as was suggested previously to explain part of a sexual shift in a protandric amphipod [18]. Generally, intersexuality in sequential hermaphrodites involves a short transition state in which an individual exhibits sex characteristics of both genders. Such sexual intermediacy may also be abnormally induced by chemicals, collectively termed endocrine disruptors. In contrast to the above-described transient intersexuality, *C. quadricarinatus*, a gonochoristic species demonstrating distinct males and females, experiences non-transient intersexuality [19]. This phenomenon has been found in both wild and cultured populations at frequencies ranging from 2–14% [20].The intersex individuals are genetic females but functional males [21], bearing an active male reproductive system and male secondary sex characters, along with a constantly arrested ovary [22], possibly due to the presence of the AG. It has been shown that upon AG ablation, intersex *C. quadricarinatus* undergo a dramatic morphological and physiological sex shift in which male reproductive organs regress, accompanied by ovarian activation and the onset of vitellogenesis [11], [23], [24]. Thus, monitoring vitellogenin, an egg yolk precursor protein, in this intersex species could serve as an accurate physiological indicator of sexual shifts [12], [24].

Although sexual shifts have been documented in invertebrates [25]–[28], the physiological mechanisms controlling these events are not fully understood. In these complicated processes, the production of gametes according to the primary gender is stopped, while the ability to produce germ cells of the opposite sex is acquired. Hence, it is probable that in such sexual shifts, the cell population of the gonad is replaced through repopulation with ‘new’ gonia, following the programmed degradation of the ‘old’ gonia via apoptosis. Indeed, apoptosis is responsible for the differentiation of the primordial gonad en route to the testis in zebrafish [29], and several anti-apoptotic factors have already been identified as being crucial for the survival of male rat germ cells [30].

The discovery of the first decapod AG-specific gene in *C. quadricarinatus*, *Cq-IAG* [15], provided a new route for studying crayfish sexuality. Still, the functionality and presumed involvement of *Cq-IAG* in sexual differentiation has yet to be determined. Gene silencing using RNAi offers one strategy to address such questions. This approach has been successful in several crustaceans [16], [31]. In the current study, we employ a functional genomics assay that relies on dsRNA silencing of *Cq-IAG* in an intersex model to demonstrate an inducible sex shift. Specifically, male external sex characters were transformed to generate animals that exhibited female maternal care-related traits, along with testicular degeneration and AG hypertrophy (hAG). In parallel, ovarian activation was observed as the result of the onset of vitellogenesis. These findings confirm the pivotal role of *Cq-IAG* in the regulation of sexual differentiation in *C. quadricarinatus*. Significantly, elucidation of the role of this gene in *C. quadricarinatus* might contribute to understanding of the evolution of the control of processes regulating sexual shifts in protandrous crustaceans and may provide unique insight into the gender-related evolution of members of the insulin superfamily.

## Results

The isolation of *Cq-IAG*, accomplished via the construction of an AG cDNA library, has been described previously [15]. In the present study, a further screening was conducted. A colony hybridization, which was used to eliminate *Cq-IAG* ESTs from subsequent sequencing, showed that 115 out of 508 colonies were positive for *Cq-IAG*. This result, showing that *Cq-IAG* comprises approximately 25% of the ESTs, is with accordance with the previous screening of the library. Out of the non-*Cq-IAG* ESTs, 177 were randomly picked and sequenced. However, no new genes were identified and thus no further screening was conducted.

**Figure 1:**
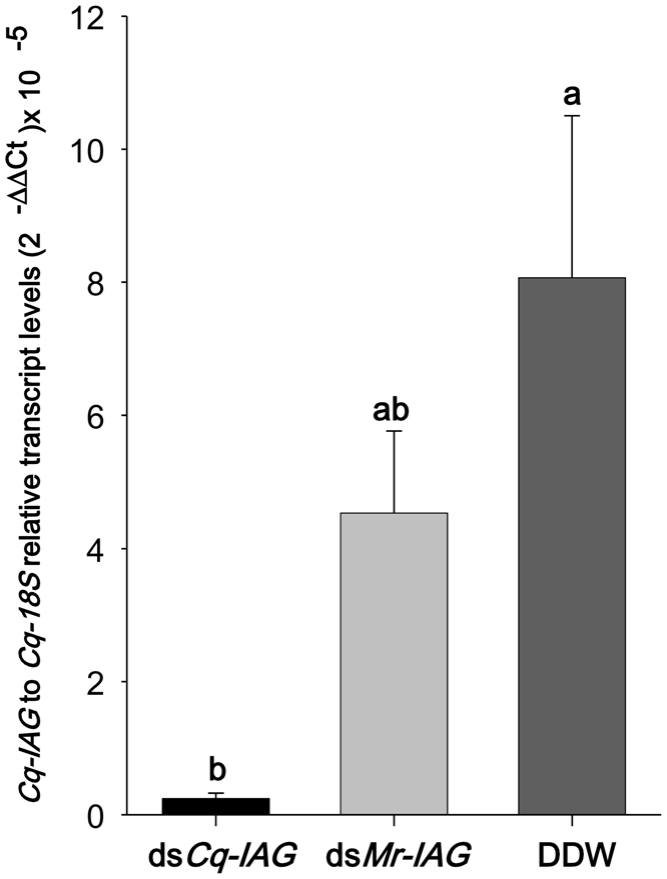
Levels of *Cq-IAG* transcripts following *in vivo* dsRNA injections in male crayfish. Relative *Cq-IAG* transcript levels were quantified in crayfish males by real-time RT-PCR following short-term silencing. Three different groups were injected with either ds*Cq-IAG* (n = 6), ds*Mr-IAG* (n = 6) or DDW (n = 6). The groups were found to be statistically different (Kruskal-Wallis statist: H (df = 2, N = 18) = 7.450, p = 0.0241). Followed by a multiple pair-wise comparison ds*Cq-IAG* group was found significantly different from the DDW (P = 0.0386) and showed a difference that turned out to be non-significant (P = 0.0798) from the ds*Mr-IAG* group. Different letters represent significant difference and error bars represent SEM.

A prerequisite for a long *in-vivo* assay demonstrating the functionality of *Cq-IAG* was to evaluate the effectiveness of gene knock-down via RNAi. For this purpose, 3 experimental groups were injected with either double stranded RNA (dsRNA) of the targeted gene, exogenous-based dsRNA or carrier only [doubly distilled water (DDW)]. The efficiency of injected *Cq-IAG* dsRNA in reducing *Cq-IAG* expression was revealed by real-time RT-PCR (Fig. 1). Specifically, the relative quantification (RQ) values noted in the experimental group injected with ds*Cq-IAG* (0.24*10^5^±0.16*10^5^) were at least 30-fold lower (Kruskal-Wallis statist: H (df = 2, N = 18)  = 7.450, p = 0.0241 followed by multiple pair-wise comparison, p<0.05) than those of the control group, which was injected with DDW (8*10^5^±2*10^5^). The exogenous *Mr-IAG* dsRNA (4*10^5^±1*10^5^) showed a difference that turned out to be non-significant (p = 0.079) from that of the ds*Cq-IAG* group, although a difference of at least one order of magnitude was observed in the raw data. This result showed that this silencing of *Cq-IAG* expression was specifically induced by *Cq-IAG* dsRNA injection, in a sequence-dependent manner.

**Figure 2:**
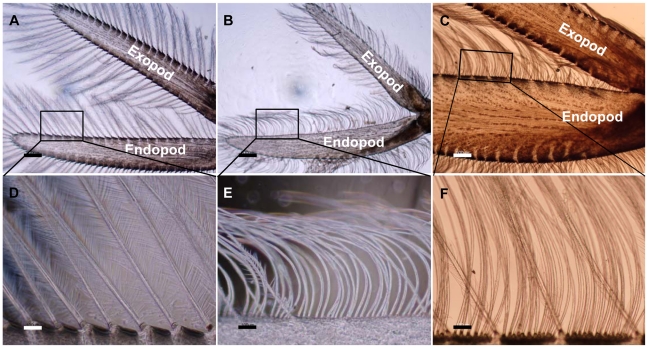
Effects of ds*Cq-IAG* injections on maternal care-related characteristics in intersex crayfish. Pleopods were collected from intersex animals injected with DDW (A) or ds*Cq-IAG* (B) and from mature females (C). Length:width ratios between endopods and exopods of DDW-injected intersex were identical (A), while those of dsRNA-injected animals (B) showed female-like biometrics. Whereas the inner side of the endopod of DDW-injected intersex bore only plumose setae (D), as in males, the inner side of the endopod of dsRNA-injected animals was lined with ovigerous simple setae (E) as is the case of mature females (F). Bottom row (bar  = 100 µm) represents an enlargement of the areas defined in squares in the top row (bar  = 500 µm).

Once the silencing effect of *Cq-IAG* dsRNA injections had been established, a long-term silencing experiment addressing *Cq-IAG* functionality was performed, with two experimental young intersex groups- dsRNA-injected and DDW-injected. Silencing of the gene in young intersex animals induced the appearance of maternal care-related secondary sex characteristics. Comparing control intersex crayfish (Fig. 2A) with dsRNA-injected animals revealed increases in the width and length of the endopodite (i.e., the internal branch of the swimming leg) [22] (Fig. 2B). The calculated endopod width index (EWI) of the dsRNA-injected group (1.44±0.130) was similar to that of characterized females, while DDW-injected intersex animals showed a significantly lower EWI (1.01±0.04, Mann Whitney U test, Z = 2.551, p<0.05), typical of males. Moreover, the male-typical plumose setae, which naturally line the inner side of the endopod of masculine intersex animals (Fig 2D) were clearly transformed in the dsRNA-injected group, which displayed the maternal-care ovigerous simple setae (Fig. 2E) normally exhibited by mature vitellogenic females (Fig. 2F).

**Figure 3:**
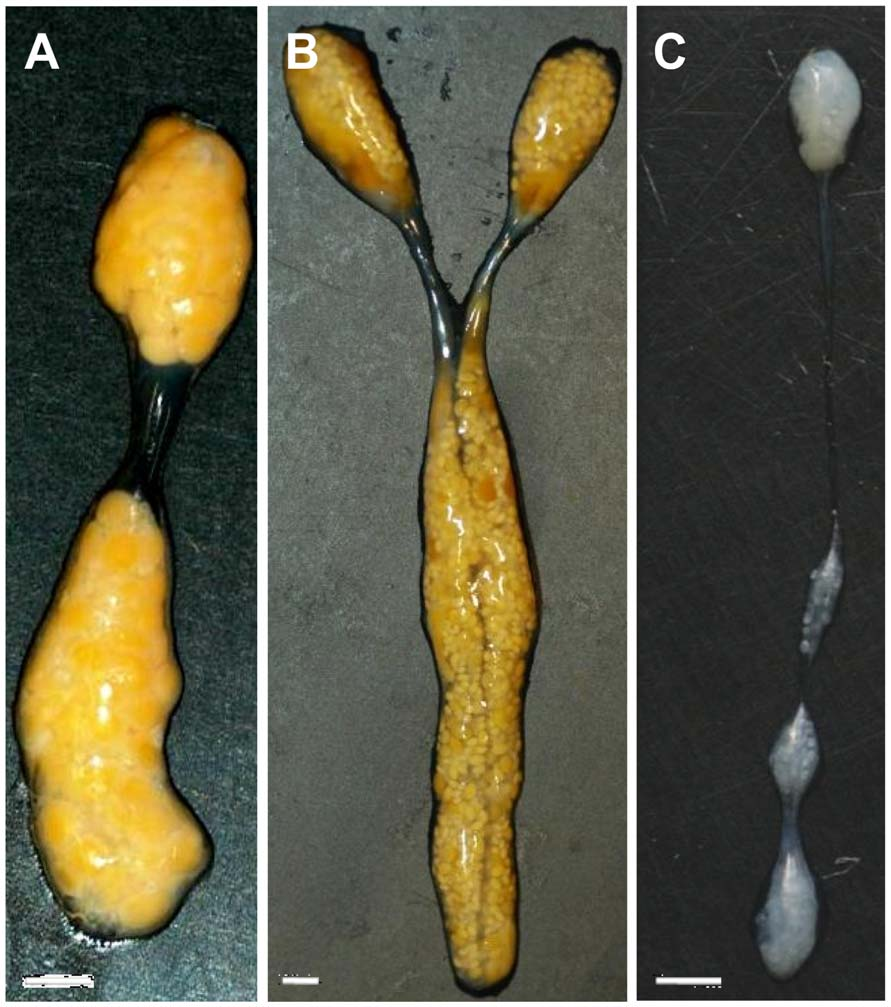
Comparison of whole ovaries obtained from dsRNA and DDW-injected intersex crayfish. The whole ovary from a dsRNA-injected intersex animal (A) contained yellowish oocytes, as in a mature female ovary (B), with an average diameter of approximately 900 µm. The ovary of a DDW-injected intersex animal (C) contained whitish oocytes, approximately 300 µm in diameter (bar  = 2 mm).

After it was shown that the pleopods of *Cq-IAG* dsRNA-injected intersex animals presented feminized characteristics, the ovaries were dissected and compared for size and coloration. The ovaries obtained from *Cq-IAG* dsRNA-injected intersex animals were larger than those of control intersex individuals (Fig. 3). Oocytes in the ovaries of *Cq-IAG* dsRNA-injected intersex animals were yellowish due to the accumulation of yolk (Fig. 3A), as normally would be observed in mature females (Fig. 3B). These oocytes were significantly larger (899±139 µm, Mann Whitney U test, Z = 2.082, p<0.05) than the whitish oocytes of control animals (Fig. 3C), with an average diameter of 305±77 µm.

**Figure 4:**
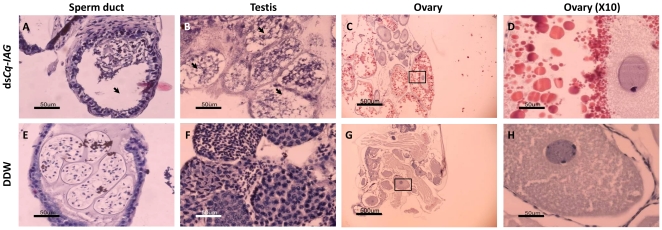
Effects of ds*Cq-IAG* injection on the reproductive system of intersex individuals. Hematoxylin- and eosin-stained cross-sections used for structure description. Components of the reproductive system of *Cq-IAG* dsRNA-injected intersex animals showed an empty sperm duct (black arrowhead, A) and inactive testicular lobules (black arrowheads, B), along with an activated ovary containing enlarged yolk-accumulating oocytes (C). A filled sperm duct (E), spermatogenic testis (F) and an arrested ovary (G) were observed in the control intersex animal. Enlarged areas within the ovaries of both groups are shown in the right hand side (D and H). Bar  = 500 µm in ovarian sections, 50 µm in sperm duct, testis and ovary, high magnification sections.

Examination of histological sections of the male reproductive system from a dsRNA-injected intersex animal showed an empty sperm duct (Fig. 4A) and degenerating testicular lobules (Fig. 4B). In these animals, there were no lobules at early spermatogenesis stages; the lobules that were present showed arrested spermatogenesis, with only few spermatozoa. In the same animals, these observations were made alongside a mature ovary (Fig. 4C) containing large oocytes, which contained yolk granules (Fig. 4C, D). In contrast, control intersex showed a sperm duct filled with a spermatophore (Fig. 4E) and large active testicular lobules at different stages of spermatogenesis, with highly abundant spermatozoa (Fig. 4F). The ovaries of the control specimens were arrested and contained small primary vitellogenic oocytes (Fig. 4G, H).

**Figure 5:**
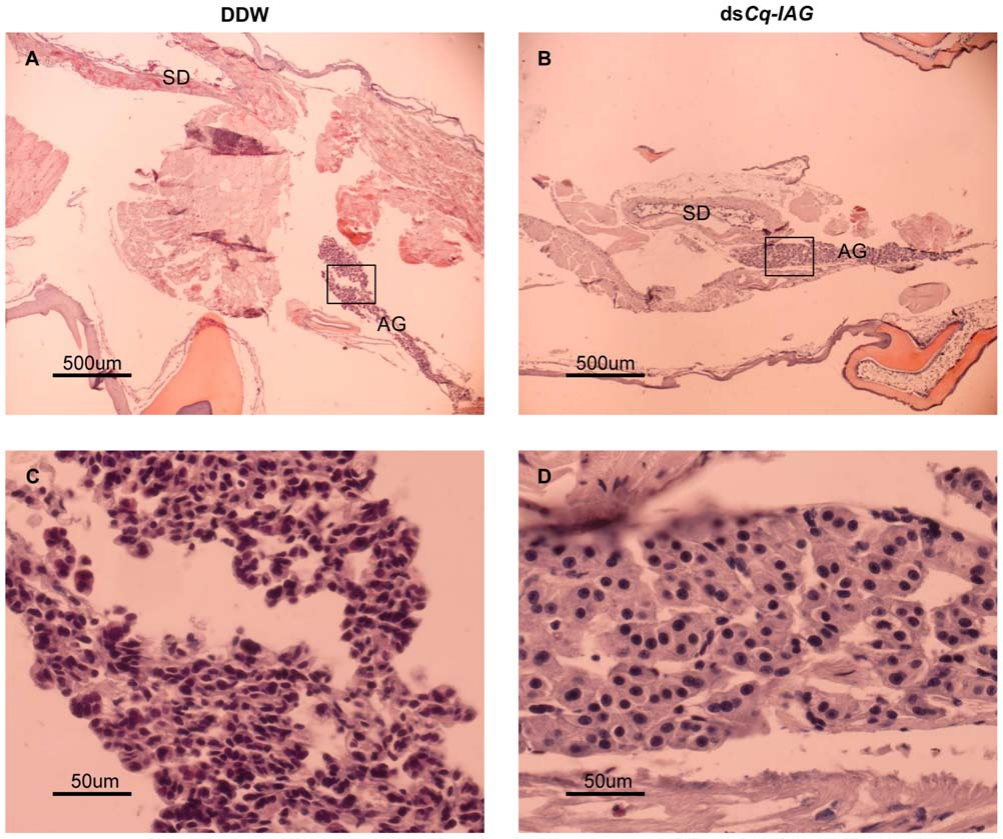
Effects of ds*Cq-IAG* injection on the androgenic gland. Sections from the base of the fifth pereiopod of DDW- (A) and *Cq-IAG* dsRNA-injected (B) intersex animals were hematoxylin- and eosin-stained. High magnification of the androgenic gland (AG) of control intersex animal (C) apparently shows smaller cells than those observed in the silenced intersex animals (D), where a hypertrophied gland comprising of apparently larger cells with highly significant larger nuclei (Mann Whitney U test, Z = 4.438, P<0.001). Top row, bar  = 500 µm; Bottom row, bar  = 50 µm. SD  =  sperm duct.

**Figure 6:**
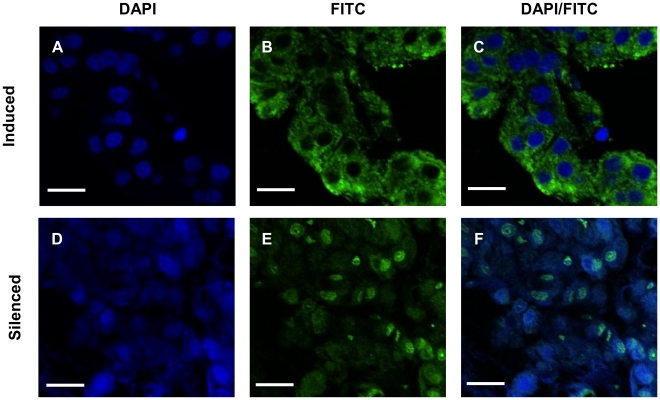
Presence of Cq-IAG in hAGs from endocrinologically-induced versus ds*Cq-IAG*-injected crayfish. Immunohistochemistry was performed on sections from the base of the fifth pereiopod of induced (top) and *Cq-IAG* dsRNA-injected (bottom) intersex animals. Large quantities of Cq-IAG, demonstrated by the green fluorescence of bound goat anti-rabbit FITC conjugated antibodies, were observed in the cytoplasm of induced AG cells (B, C). Reduced levels of the Cq-IAG hormone were observed in the cytoplasm of AG cells of ds*Cq-IAG* -injected intersex animals (F). DAPI counterstain was used to identify nuclei in both induced (A, C) and silenced (D, F) intersex animals. Bar  = 20 µm.

Histological examination of the morphology of the AG in the *Cq-IAG* dsRNA-injected group showed arrest of the male reproductive system and activation of female sexual morphology (Fig. 4), accompanied by hypertrophy of AG cells, with significantly larger nuclei (7.58±0.16 µm, Mann Whitney U test, Z = 4.438, p<0.001) (Fig. 5D), than in the control group (5.67±0.22 µm) (Fig. 5C). The AG cells in the treatment group also appeared to be lager in their total size. AG hypertrophy in the dsRNA-injected group differed markedly from that induced by ablation of the X-organ sinus-gland complex in the eyestalk [10], [32], in which massive production of the Cq-IAG hormone takes place within the AG cells (Fig. 6B, C), possibly due to the removal of a specific inhibiting agent. On the contrary, AG cells of the hypertrophied *Cq-IAG*-dsRNA-injected intersex were characterized by low levels of the Cq-IAG hormone (Fig. 6E, F), a possible consequence of the RNAi application.

**Figure 7:**
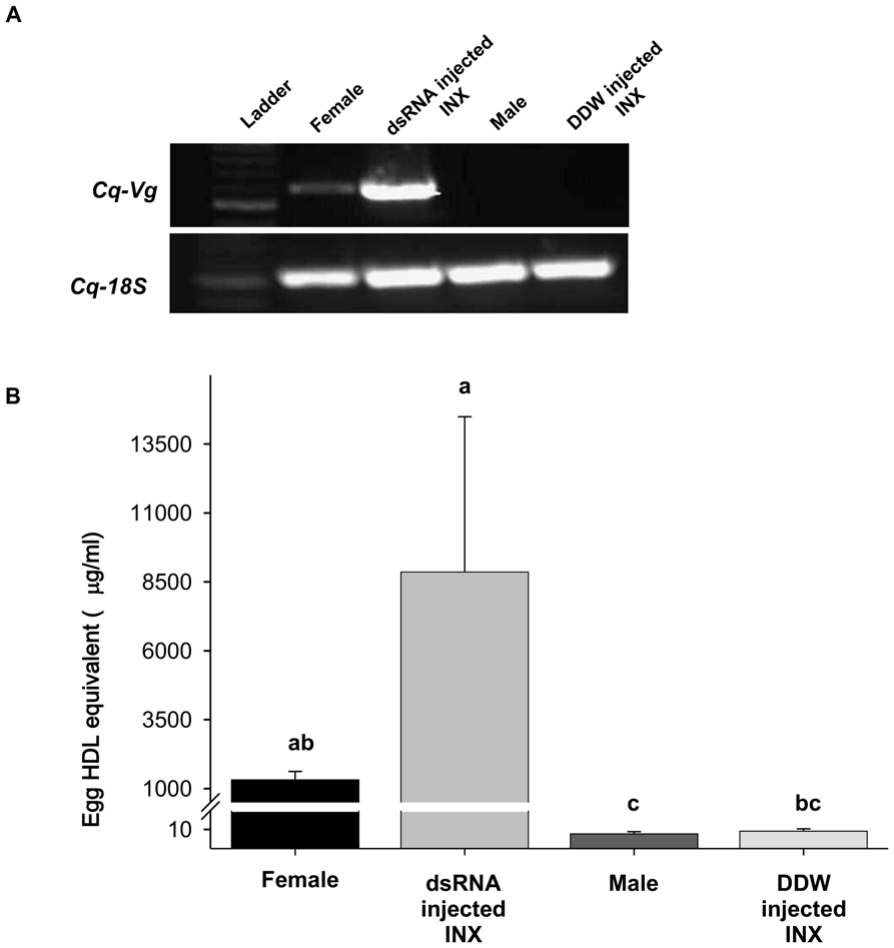
Levels of vitellogenin and expression of its encoding gene following *Cq-IAG* silencing in intersex crayfish. (A) *Cq-Vg* transcription in the hepatopancreas was demonstrated by RT-PCR. A single band was observed in the ds*Cq-IAG*-injected intersex animal sample but not in the control intersex sample. Samples of a vitellogenic female and a male served as controls. (B) Detection of the vitellogenin protein in the hemolymph was conducted by ELISA using anti-Cq-Vg antibodies. Hemolymph samples were collected from DDW-injected intersex (INX) animals (n = 7), ds*Cq-IAG*-injected INX (n = 3), vitellogenic females (n = 6) and mature males (n = 6). Egg high density lipoprotein (HDL) equivalent levels in dsRNA-injected INX were similar to those of vitellogenic females (Kruskal-Wallis statist: H (df = 3, N = 22) = 16.137, p = 0.001 followed by multiple pair-wise comparison). Negligible levels of egg HDL equivalents were detected in control intersex and mature males. Different letters represent significant differences (p<0.05±SEM).

During vitellogenesis in mature female *C. quadricarinatus*, the *vitellogenin* (*Cq-Vg*) gene is transcribed in the hepatopancreas, and its translated product is mobilized through the hemolymph to the ovaries, where it accumulates. *Cq-IAG* dsRNA injection induced both *Cq-Vg* transcription and expression of its encoded yolk protein (Fig. 7). *Cq-Vg* expression was demonstrated by RT-PCR (Fig. 7A) as a single band when amplified cDNA from the hepatopancreas of dsRNA-injected intersex individuals served as the template. Hepatopancreatic cDNA samples of vitellogenic females and mature males served as positive and negative controls, respectively. The presence of cDNA was ensured by amplifying the *C. quadricarinatus 18S rRNA* (*Cq-18S*) housekeeping gene, which served as control for RNA extraction and the RT reaction. The 106-kDa vitellogenin polypeptide, indicative of secondary vitellogenesis, was detected and quantified in the hemolymph by using ELISA (Fig. 7B), showing similar levels in males (7±1 µg/ml) and control intersex animals (9±1 µg/ml) (Fig. 7B). In contrast, hemolymph samples obtained from dsRNA-injected intersex animals showed a significant increase in the level of yolk proteins (8860±5629 µg/ml, Kruskal-Wallis statist: H (df = 3, N =  22) = 16.137, p = 0.001 followed by multiple pair-wise comparison, p<0.05) reaching a concentration even higher than that seen in vitellogenic females (1315±308 µg/ml) (Fig. 7B).

## Discussion

While previous studies have demonstrated the influence of the AG on a wide array of characteristics related to sexual development and growth of *C. quadricarinatus* [10], [12], only recently a specific AG factor accounting for the male phenotype in this species has been suggested with the identification of *Cq-IAG*, the first insulin-like AG specific gene to be revealed in decapods [15]. Screening of an AG-derived cDNA library revealed that *Cq-IAG* comprises about 26% of the ESTs in the library, possibly hinting at the importance of this gene in AG function.

*Cq-IAG* belongs to the insulin-like superfamily of genes, which are generally assigned to processes of metabolism and growth. However, some insulin growth factors are also linked to differentiation and apoptosis [33]. The rare case of *Cq-IAG* and several other AG-specific insulin-like genes, which are expressed in a gender-specific manner restricted to males, has been thus far recorded exclusively in Crustacea [13]–[16]. Sequence conservation among crustacean AG-specific insulin-like peptides is relatively low (approximately 16–29%), considering their putative central role as male sex hormones. However, their predicted structures are quite similar and conserved, comprising heterodimeric peptide chains, resulting from the proteolysis of the pro-hormone. Moreover, the various versions of the peptide share the exact same positions of cysteine residues, which govern folding via the formation of disulfide bonds. Our results regarding the induction of femaleness, combined with the demasculinization of male prawn *M. rosenbergii* [16], both achieved by the silencing of AG-specific insulin-like genes, indicate that insulin-like genes have evolved not only in the context of metabolism and growth but also in the context of male sex differentiation. However, in substantial difference from the prawn, the present study, addressing an intersex crayfish, suggests that in addition to the induction of maleness, the AG-specific insulin-like factor serves as a gender switcher that controls the maleness/femaleness balance in an intersex organism. This concept gives rise to a hypothesis linking AG-specific insulin-like factors to the regulation of sexual shifts in crustacean sequential hermaphrodites. Although insulin-like genes have been documented in the context of insect sexuality [34], proliferation and growth [35], [36], no evidence of a gender-specific insulin-like gene has previously been recorded in this taxonomic group. The fact that such a gender-specific insulin-like gene, playing a pivotal role in crustacean sexual differentiation, does not exist in insects is somewhat puzzling, since crustaceans are considered to be evolutionarily older than insects [4].

An important observation of the present study is the extensive AG hypertrophy seen following *Cq-IAG* silencing, a procedure that led to low transcription and poor production of the proteinaceous hormone encoded by the *Cq-IAG* gene. It is thus suggested that the AG-specific insulin-like factor Cq-IAG may, therefore, regulate its own production and secretion by means of feedback inhibition. Thus, the hybridization of *Cq-IAG* mRNA with the exogenous *Cq-IAG* dsRNA, which led to the degradation of the corresponding mRNA, permitted only basal levels of protein production and possibly induced compensation, leading to hypertrophy. We cannot rule out the possibility that higher hierarchy levels of regulation (e.g. paracrine and/or endocrine) could possibly also regulate AG activity, thereby inducing the observed hypertrophy, since hypertrophy of a gland in compensation for low levels of secreted product is well documented in cases of endocrine deficiencies [37].

This study rests on the sexual plastic model of intersexuality as the basis for elucidating the role of the AG-specific insulin-like gene in *C. quadricarinatus* and its involvement in sexual regulation. *Cq-IAG* silencing in the intersex crayfish clearly revealed the involvement of this gene in maintaining maleness, as reflected in the empty vas deference and arrest of spermatogenesis that resulted from knock-down of *Cq-IAG* levels. Moreover, the extensive degeneration of the testicular lobules observed indicates that Cq-IAG is essential for male germ cell survival. Such involvement of an insulin-like peptide in testicular germ cell survival has also been documented in rats [30]. Moreover, the effect of Cq-IAG in extending the viability of male germ cells is similar to that seen with the mammalian male sex hormone, testosterone [38], under similar circumstances. Such resemblance implies that Cq-IAG acts in an androgenic way, being required for male gonad differentiation and ongoing spermatogenesis. Importantly, in silenced intersex *C. quadricarinatus* individuals, male regression occurred simultaneously with ovarian activation and the onset of vitellogenesis, suggesting that a prerequisite for the activation of femaleness is a lack of sufficient levels of the AG factor, which in turn leads to apoptosis and degeneration of the male gonad and sexual shift. The results of this study, which are in accordance with previous ones [16] and which demonstrate the effect of an insulin-like gene on male sexuality, are based on loss of function experimentation. Moreover, in a gain of function study using a recombinant protein based on the orthologous AG-specific insulin-like gene in females of the isopod *A. vulgare* [39], the induction of masculinity was documented, thus suggesting a direct role for this AG family of genes in governing masculinity. Such a direct effect of an AG-specific insulin-like peptide using recombinant proteins has not been shown in decapods as yet. Due to the lack of such direct evidence, secondary/indirect regulation of masculine differentiation by an AG-specific insulin-like peptide cannot be ruled out.

The sexual shift induced in this study upon manipulation of a single AG gene may provide, for the first time, insight into the mechanisms underlying sexual shifts that occur naturally in many sequential hermaphrodites in the animal kingdom [40], particularly in protandric hermaphrodite crustaceans. While the endocrine mechanism underlying this phenomenon is not yet known, our results suggest that in protandric hermaphrodite crustaceans, a single insulin-like gene is involved in the regulation of the sexual shift. Our findings also allow us to posit that such an insulin-like gene sustains maleness in early life, followed by intrinsic silencing later on, which triggers sex inversion towards femaleness via an intersexual transition state.

The building of a comprehensive picture of the processes of natural sexual shift opens the window for study of abnormal processes of sexuality. Such cases can be induced by man-made environmental pollutants that act as endocrine disruptors [41]. How these substances induce sex abnormalities remains to be elucidated. Thus, addressing mechanisms in naturally occurring sequential hermaphroditism, such as the AG and the secreted insulin-like peptide studied here, could serve to elucidate pathways inducing sex abnormalities in crustaceans, with possible extrapolation to other arthropods. Such insight carries significant applied implications, such as in the possibility of creating non-breeding, all-male crustacean populations [42] via AG-specific insulin-like gene manipulation. Given that males grow larger than females in some commercialized species, the implications of this possibility are clear.

## Materials and Methods

### AG cDNA library screening

The previously constructed *C. quadricarinatus* AG cDNA SSH library [15] was further screened. To avoid *Cq-IAG*-repeated sequencing, four plates containing 127 colonies each (each cloned with a single EST from the described library) were screened, using a colony hybridization method [43] with a radio-labeled probe. Briefly, a *Cq-IAG* cDNA probe was synthesized using a random priming labeling mix (Biological Industries). The probe was hydrolyzed to reduce its length to ∼200 b, as described in the DIG Application Manual (Roche Applied Science). The hydrolyzed probe mix was then hybridized to clones that had been fixed to nitrocellulose membranes. The membranes were hybridized over night, washed, sealed and exposed to BioMax MS Kodak film as described [43]. The films were then developed according to supplier's instructions. Subsequently, 177 colonies that were considered as non-*Cq-IAG* were picked to liquid LB, grown over night (Qiagen DirectPrep 96 Miniprep) and sequenced as previously described [15].

### Animals

Intersex *C. quadricarinatus* animals (each weighing 1–5 g and having a single male genital opening and two female genital openings [21], [22]) were grown and maintained in circular cages 10 cm in diameter, floating in 40×40×50 cm^3^ aquaria situated at Ben-Gurion University of the Negev. Young males (15–17 g), mature males and females (40–70 g) of that species were also maintained in aquaria of the same size. The mature males had been subjected to surgical removal of the X-organ sinus-gland, an endocrine manipulation causing hypertrophy of the AG, as previously described [10], [32]. The water temperature was kept at 28°C±2, and water quality was assured by circulating the system's total volume through a biofilter. A photoperiod of 14L: 10D was applied. Food, comprising shrimp pellets (Rangen Inc., 30% protein), was supplied *ad libitum* three times a week. Animals' weights were measured using analytical scales with a ±0.01 g error.

### dsRNA preparation

The pGEM-T easy vector (Promega Corp., Madison, WI) including the *Cq-IAG* open reading frame (ORF) (accession no. DQ851163) was digested alternatively by NdeI and XbaI restriction enzymes (New England Biolabs Inc. Ipswich, MA), thereby yielding linear templates for sense and antisense *Cq-IAG*, respectively. Upon digestion, a small aliquot of each template was examined for digestion efficiency on a 1.3% agarose gel; linearized vectors were purified by the standard phenol:chloroform protocol and ethanol precipitation. Single-stranded RNAs were synthesized based on the above-mentioned linearized plasmids with a MEGAscript T7 kit (Ambion, Inc., Austin, TX) according to the manufacturer's instructions. Sense and antisense RNA were purified and hybridized as described previously [44]. Control dsRNA for a short-time *in-vivo* assay was synthesized based on the ORF of *Mr-IAG*, as previously described [16]. Although *Mr-IAG* encodes an orthologous peptide, its nucleotide sequence shows no significant similarity to *Cq-IAG* (BLAST algorithm-“align two sequences”); thus, it was chosen as a negative dsRNA control.

### In-vivo injection of dsRNA

For a preliminary short-term silencing experiment, young male crayfish (15–17 g) were divided into three treatment groups, as follows: *Cq-IAG* dsRNA-injected (n = 6), *Mr-IAG* dsRNA-injected (n = 6) and DDW-injected (n = 6). The dsRNA-injected animals were injected with 2 µg of dsRNA/g animal into the sinus of the fifth walking leg [44], and the DDW-injected group received an equivalent volume of DDW. Injections were given once a day on two consecutive days followed on the third day by dissection of the animals, to isolate the AGs.

For the long-term silencing experiment, a group of 15 intersex individuals (each weighing 1–5 g) were selected and assigned into two groups: ds*Cq-IAG*-injected (n = 8) and DDW-injected (n = 7). The same procedure as the preliminary experiment was applied for the long-term assay, with the exception that the injections were given biweekly over a period of 25–30 weeks (2–5 molts from the beginning of the *in-vivo* assay).

### RNA extraction and real-time RT-PCR

RNA was extracted from the AGs of males used in the short-term preliminary *in-vivo* experiment. Total RNA was isolated with the EZ-RNA Total RNA Isolation Kit, used according to the manufacturer's instructions (Biological Industries, Beit Haemek, Israel). First-strand cDNA was synthesized by means of reverse transcriptase reaction using the Verso™ cDNA Kit (Thermo Fisher Scientific Inc.) with 1 µg of total RNA. RQ of *Cq-IAG* transcript levels were obtained using the following primers: IAG qPCR_F: 5′-GGCCTCCTCCCCTATCTGT-3′ and IAG qPCR_R: 5′-CCAGCCAGCAGCAGAATAGT-3′ with the FastStart Universal Probe Master (Rox) (Roche Diagnostics GmbH) and Universal ProbeLibrary Probe #144 (Roche). *Cq-18S* (accession no. AF235966), which was used as a normalizing gene, was also quantified by means of real-time RT-PCR using the primers: q*Cq-18S*_F: 5′- CTGAGAAACGGCTACCACATC-3′ and q*Cq-18S*_R: 5′-GCCGGGAGTGGGTAATTT-3′ with the above-mentioned mix and Universal ProbeLibrary Probe #74 (Roche). Reactions were performed with the ABI Prism 7000 Sequence Detection System (Applied Biosystems, Foster city, CA, USA).

### RT-PCR

RNA was extracted from samples of hepatopancreas and AG from mature males, from samples of hepatopancreas from mature females, and from the hepatopancreas and AG of the long-term *in vivo* experimental animals, as mentioned above. The RT reaction was performed using M-MLV H minus reverse transcriptase (Promega), with 1 µg of total RNA from each sample according to the manufacturer's instructions. *Cq-Vg* (accession no. AF306784) was amplified by means of PCR with REDTaq ReadyMix™ PCR Reaction Mix (Sigma; one cycle at 94°C–3 min; 37 cycles at 94°C–30 s, 58°C–30 s, 72°C–1 min; one cycle at 72°C–10 min) with the forward primer Cq-Vg F: 5′- AACGAGAGCCAGTCTTTGTGGCTG -3′ and the reverse primer Cq-Vg R: 5′- CAGCTTGTAGCTGTATGGACTACCAAG -3′ using cDNA obtained from hepatopancreas. *Cq-18S* served as the internal control for the RNA extraction and RT reaction. PCR products were separated on 1.3% agarose gel with Tris-acetate EDTA buffer, and bands were documented. AG cDNA was used to amplify *Cq-IAG* by PCR (one cycle at 94°C–3 min; 35 cycles at 94°C–1 min, 55°C–45 s, 72°C–50 s; one cycle at 72°C–10 min) with the reaction mix described above, using adaptor primers containing a gene-specific sequence flanked by a restriction recognition site and a T7 RNA polymerase sequence. The primers used were the forward *T7*-NdeI- F IAG: 5′- *TAATACGACTCACTATAGGG* TCTAGACTGATTGACTTCGACTGTGG -3′ and reverse *T7*-XbaI- R IAG: 5′-*TAATACGACTCACTATAGGG* CATATGAACTGACGTAGATTCCGTCC-3′. PCR products were separated as mentioned above. A fragment was excised, purified (Invisorb Spin DNA Extraction, Invitek, Berlin, Germany) and cloned into the pGEM-T easy vector (Promega). Clones containing the insert were isolated, and plasmid DNA was purified using HiYield Plasmid Mini Kit (RBC Bioscience, Taiwan).

### Histological preparations

The reproductive systems of the dsRNA-injected and DDW-injected intersex animals were dissected for morphological observation. Sperm ducts, testes, ovaries and AGs were fixed, dehydrated, embedded in paraffin and sectioned as previously described [16]. Sections were stained hematoxylin and eosin and observed under a light microscope.

### Immunohistochemistry

For the immunodetection of Cq-IAG, a specific antibody was generated as follows: Briefly, a recombinant Cq-IAG peptide was expressed in *Escherichia coli* cells, purified using a nickel column (Ni-NTA Superflow Cartridge, Qiagen) and validated by MALDI-TOF. The confirmed recombinant Cq-IAG peptide was injected into a rabbit. The specificity of the antiserum was demonstrated by crossreactivity with the recombinant Cq-IAG by means of Western-blot. For the immunohistochemistry, paraffin sections of AGs obtained from endocrinologically induced [10] and dsRNA-injected intersex animals were deparaffinized and rehydrated. Sections were then incubated in citrate buffer (0.5 M, pH 6, 30 min in 95°C) for antigen retrieval and washed in phosphate-buffered saline (PBS) (10 mM, pH 7.4). Blocking (2% normal goat serum, 0.1% Triton X 100, 0.05% Tween 20 in PBS) lasted for 1 h at room temperature, followed by incubation with the primary Cq-IAG antibody (1∶10,000). Slides were washed in PBS and incubated with a secondary goat anti-rabbit FITC conjugate antibody (1∶500 in PBS with 0.2% fish skin gelatin) for 1 h at room temperature. After PBS washes, slides were mounted (DAPI 1∶1000 in PBS and 50% glycerol) and imaged using a confocal microscope.

### Anatomical, morphological and physiological measurements

The first left pleopod was removed from all intersex individuals at the beginning of the experiment, and the newly regenerated pleopod was removed at the end of the experiment. The widths of the endopod and the exopod were measured to calculate the EWI [22]. The type of setation on the inner side of the endopod was observed by using a light microscope with an objective micrometer. The average diameter of 15 randomly selected oocytes from each ovary was determined under a light microscope with an objective micrometer (x40, ±25 µm). Similarly, the average diameter of 15 randomly selected nuclei of cross-sectioned silenced and control AGs were also determined (x1000, ±1 µm). Vitellogenesis levels were quantified by ELISA [23] using an anti secondary-vitellogenic-specific 106-kDa polypeptide antibody on hemolymph samples collected from dsRNA-injected intersex animals (n = 3), DDW-injected intersex animals (n = 7), mature females (n = 6) and mature males (n = 6).

### Statistical analyses

Data are expressed as means ± SEM. Due to the nature of the experiment the analyzed groups were relatively small and not normally distributed. Thus, non-parametric tests had to be used. All statistical analyses of a single parameter compared between 2 groups (e.g. EWI, oocytes diameter and AG nuclei diameter) were analyzed using the Mann Whitney U Test. The three and four groups analyzed in the real time RT-PCR and ELISA experimentation, respectively, were analyzed using the Kruskal Wallis Test followed by the correction of a multiple pair-wise comparison (built-in within the STATISTICA software) as accepted. We took into account that some groups might differ only in a marginally significant manner due to the usage of a multiple non-parametric test, which relies on a ranking principle.
